# Supplementary material for: Capitulum Development and Gametophyte Ontogeny: Histological Insight into the Reproductive Process of a Hexaploidy Population of Solidago canadensis in China
Source: Plants (Basel). 2022 Aug 8;11(15):2073. doi: 10.3390/plants11152073 (PMC9370182; doi:10.3390/plants11152073)
Supplement: Supplementary file 1 [file plants-11-02073-s001.zip › plants-1761983-supplementary/Supplementary Files/File S1 - Schedule of Experiments.pdf]

1. 30 Nov 2005 **Seed collection:** seeds from 10 plants (ramets about 10m from each other) in the population (Population number: CN04; Location: E: 118° 53' 25", N: 32° 01' 25") were collected.
2. 05 Dec 2006 **Germination experiments:** About 500 Seeds were germinated in seedling trays filled with a 1:2 mixture of soil and sterilized compost in a greenhouse.
3. 06 March 2007 **Transplanted in a 5L plastic pots:** 210 small plants (3–5 leaves) were selected and transplanted in a 5L plastic pots (3 plants per pot) with the same growing medium and were grown in a netted hoop house.
4. 01 Sept to 10 October 2007 **Capitulum sampling:** plants at the same growth stage were selected every day and 1 top capitulum was sampled from each of 20 randomly selected individual plants. The lengths of the capitula were measured. In order to record morphological variations of the capitulum during capitulum development, 10 capitula were peeled off with a dissecting needle and a scalpel to remove the periclinium, double-fixed with glutaraldehyde and osmic acid. The other 10 capitula were fixed with FAA fixative. For later observation experiments.
5. 05 Nov 2007 to 10 Jan 2008 **Microscopy observation experiments**
6. 01 Sept to 10 October 2008 **Capitulum sampling:** same as experiments in 2007.
7. 01 Nov 2008 to 20 Dec 2008 **Microscopy observation experiments**
